# Oxidative Stress in Endurance Cycling Is Reduced Dose-Dependently after One Month of Re-Esterified DHA Supplementation

**DOI:** 10.3390/antiox9111145

**Published:** 2020-11-18

**Authors:** Lydia de Salazar, Carlos Contreras, Antonio Torregrosa-García, Antonio J. Luque-Rubia, Vicente Ávila-Gandía, Joan Carles Domingo, Francisco Javier López-Román

**Affiliations:** 1 Sports Physiology Department, Faculty of Health Sciences, UCAM Universidad Católica San Antonio de Murcia, 30107 Guadalupe (Murcia), Spain; dssflydia@ucam.edu (L.d.S.); ajluque@ucam.edu (A.J.L.-R.); vavila@ucam.edu (V.Á.-G.); jlroman@ucam.edu (F.J.L.-R.); 2 Innova, Health and Sport Institute, 30100 Murcia, Spain; cjcontreras@ucam.edu; 3 Department of Biochemistry and Molecular Biomedicine, University of Barcelona, 08007 Barcelona, Spain; jcdomingo@ub.edu; 4 Biomedical Research Institute of Murcia (IMIB-Arrixaca), 30120 Murcia, Spain

**Keywords:** DHA, Docosahexaenoic acid, omega-3, PUFA, antioxidant, exercise, oxidative stress, re-esterified triacyclglycerols

## Abstract

Docosahexaenoic acid (DHA) supplementation can reduce exercise-induced oxidative stress generated during long aerobic exercise, with the minimum dose yet to be elucidated for physically active subjects. In this study, we performed a dose finding with re-esterified DHA in triglyceride form in a randomized double-blind parallel trial at different doses (350, 1050, 1750, and 2450 mg a day) for 4 weeks in males engaged in regular cycling (*n* = 100, 7.6 ± 3.7 h/week). The endogenous antioxidant capacity of DHA was quantified as a reduction in the levels of the oxidative stress marker 8-hydroxy-2′-deoxyguanosine (8-OHdG) recollected in 24-h urine samples after 90 min of constant load cycling before and after intervention. To ascertain incorporation of DHA, erythrocyte polyunsaturated fatty acid (PUFA) composition was compared along groups. We found a dose-dependent antioxidant capacity of DHA from 1050 mg with a trend to neutralization for the highest dose of 2450 mg (placebo: *n* = 13, F = 0.041; 350 mg: *n* = 10, F = 0.268; 1050 mg: *n* = 11, F = 7.112; 1750 mg: *n* = 12, F = 9.681; 2450 mg: *n* = 10, F = 15.230). In the erythrocyte membrane, the re-esterified DHA increased DHA and omega-3 percentage and decreased omega 6 and the omega-6 to omega-3 ratio, while Eicosapentaenoic acid (EPA) and PUFA remained unchanged. Supplementation of re-esterified DHA exerts a dose-dependent endogenous antioxidant property against moderate-intensity long-duration aerobic exercise in physically active subjects when provided at least 1050 mg a day for 4 weeks.

## 1. Introduction

Omega-3 have been studied for its health benefits during the last decades [1,2]. They represent a broad family of polyunsaturated fatty acids (PUFAs), with the most active compounds probably being the double bond molecules EPA (Eicosapentaenoic acid, C20:5*n-3*) and DHA (Docosahexaenoic acid, C22:6*n-3*), which exhibit different biochemical behaviors when administered [3,4,5], thus they should not be interpreted as the same intervention treatment [6,7]. There is some controversy about the prooxidant/antioxidant capacity of DHA since its six double bonds are susceptible to oxidation and in fact, it showed a dual effect both as a pro-oxidant and antioxidant when given at different doses [8]. Oxidation by-products of DHA contribute to increased oxidative stress in the organism, but on the other side, they contribute to the expression of endogenous enzymatic antioxidant systems, like catalase, superoxide dismutase, and glutathione peroxidase [9,10,11,12,13,14].

The minimum DHA supplementation to elicit this effect remains inconclusive, partly due to the diversity of protocols employed in the literature, finding no variation in redox status in some population samples [15,16,17], and with positive results for other samples studied [18,19,20]. Additionally, clinical trials employed different fish oil products, which are not equally bioavailable [21], and dosing strategies that involve different cumulative doses and intervention times. Aerobic exercise inevitably augments oxygen consumption and increases acute oxidative stress [22,23] whose magnitude is dependent on the intensity and duration, as well as the training status of the subject whose antioxidant defense system is more adapted [24]. Thus, a model that employs modest-intensity and long-duration aerobic exercise in trained subjects can serve as a model to assess the antioxidant capacity of DHA at different doses. One gap we found in the literature is that the impact of DHA on oxidative stress in healthy physically active people has not been studied before, thus results from this assessment would serve as a reference to healthy subjects engaged in regular exercise.

In the present study, we conducted a dose finding of a high content (>70% of total fatty acids) of re-esterified DHA (rDHA) to assess the endogenous antioxidant effect in moderate-intensity exercise-induced oxidative stress in healthy active people. We hypothesized a response in a dose-dependent manner, with a greater effect for higher doses.

## 2. Materials and Methods

### 2.1. Trial Design

A double-blind, parallel, unicentric, controlled, and randomized experimental study was employed, with five different study arms to test the effect of DHA supplementation (rDHA at four different doses: 350, 1050, 1750, or 2450 mg) or placebo (PLA) on the oxidative stress generated by a moderate-intensity long-duration cycling test. Randomization was performed by a scientist not participating in the study, using software (Epidat 4.2, 2016) that generated random codes, which were assigned to participants. An initial incremental exercise test to exhaustion (IETE) was carried out to make an initial assessment of the physical conditioning of each participant. Then, the supplementation protocol of each group (rDHA or PLA) commenced for a period of 4 weeks, after which cyclists underwent the same exercise protocol to measure the intervention effect.

### 2.2. Subjects

Inclusion criteria were as follows: (1) Healthy male adults engaged in regular cycling of at least 5 h per week. Exclusion criteria were as follows: (1) serious clinical pathology or antecedents; (2) regular smoker; (3) regular consumer of alcoholic beverages; (4) supplementation with omega 3 fatty acids or food enriched in omega-3 fatty acids in the last 4 weeks; (5) subjects who had undergone ionizing radiation for accidental, diagnostic, or therapeutic reasons during the trial or in the month prior to it; and (6) allergy to fish or any of its by-products. Participants were informed (verbally and written) of the purpose of this study, the characteristics of the product used for supplementation, its effects, as well as any possible risk and side effects resulting from the supplement and the procedures of the study. Subjects were informed of their right to quit the study at any time, without the need to provide any reason. Participants gave written consent before the study was started. The study protocol and informed consent were approved by the Ethics Committee of the Catholic University of Murcia (UCAM protocol number 21052008) and were in agreement with the Declaration of Helsinki. A total of 75 subjects were finally recruited for the study.

### 2.3. Supplementation Protocol and Dietary Assessment

The study population was divided into five groups, based on daily re-esterified triacylglycerol (rTAG) rDHA (BRUDY PLUS^®^, BRUDYTECHNOLOGY, Barcelona, Spain) consumption or PLA (refined sunflower oil) provided by the same manufacturer. Re-esterified triacyclglycerols are concentrated fish oils in which the natural TAGs are transferred to ethanol-forming etyl-esters and, after removal of undesired FAs, reconverted into triacylglycerols (thus re-esterified triacyclglycerols: rTAG). The composition per soft-gel of rDHA was: DHA (Tridocosahexaenoine-AOX^®^) 350 mg, EPA 40 mg, total Omega-3 content 405 mg, and a total fatty acid content of 500 mg, consumed in a single dose in the morning before breakfast, for 4 weeks. Participants were allocated to one of the following five groups: (1) PLA (3 softgels); or rDHA: (2) 350 mg/day (1 soft-gel); (3) 1050 mg/day (3 soft-gels); (4) 1750 mg/day (5 soft-gels); or (5) 2450 mg/day (7 soft-gels). Both products were identical in appearance: soft-gel oval, with transparent gelatin and yellow-colored oil, inside a plastic container, properly labeled and identified to preserve blinding. The batch number was the only difference between them. Sunflower oil is not a true placebo since it contains tocopherols with antioxidant properties [25]. Refined sunflower oil has a decreased amount of tocopherols by up to a third [26] but can still exert a biological effect. For simplicity, we will refer to it as a placebo in the paper, even if this is not an accurate description. Participants were asked to return the empty packs to ensure compliance by counting the left-over soft-gels. For the follow-up, participants were reminded verbally to consume the product.

Dietary assessment was performed through a qualitative and quantitative questionnaire that subjects had to fill out during the study period, which was analyzed by a nutritionist to determine dietary intake using software (Cronometer Software Inc., https://cronometer.com). Additionally, subjects were instructed in products that should not be consumed at breakfast prior to the physical test (products with antioxidant properties) (see Appendix A, Table A1), which could interfere with the reading of the primary outcome.

### 2.4. Exercise Test

Each subject performed an initial physical assessment to determine the physical condition of the subjects and the external load of the second test. The initial assessment consisted of an incremental exercise cycling test to exhaustion (IETE) while the main test consisted of a square-wave endurance exercise test (SWEET) to induce exercise oxidative stress. Both tests were spaced 1 week apart to allow full physical recovery. A second SWEET test was performed after intervention to quantify the intervention effect in each group. In every occasion, they had to arrive after a 2-h fast and bring their own bike. All tests were conducted in the morning at the same time, and they were instructed not to change their physical activity habits during the study and to avoid physical exercise 48 h before the initial assessment, and 24 h before the main tests. Environmental conditions (room temperature and humidity) were replicated in every exercise test for optimal conditions using a room air conditioning system.

#### 2.4.1. Initial Physical Assessment: Aerobic Capacity and Health Assessment

A preliminary IETE test was performed to assess the training status of volunteers at baseline and establish the load to be employed in the SWEET. Volunteers performed an incremental exercise to exhaustion using their own personal bike mounted on an electrically braked ergometer. The starting load was 12 km/h with load increases of 2 km/h every minute, maintaining a constant slope of 2%. A self-selected cadence between 60 and 100 revolutions per minute had to be maintained during the whole test. The following measures were employed to ensure repeatability: (1) Front-rear slope-ratio was corrected to zero (using a front wheel riser); (2) bike configuration (gear set, saddle and handlebars) must be kept during the study; (3) Bike fitting (seat-post height and angle, handlebar reach, height and grip position) must be the same; and (4) the preferred pedaling system (use of cycling shoes and type of clip/cleat) should be consistent. Exhaustion was deemed to occur when the subject decided to stop, when pedal cadence dropped 20 RPM below the minimum cadence established (i.e., 40 RPM), or when power output could not be maintained. During the test, volunteers were verbally encouraged by the staff to exert maximal effort.

During the entire test, subjects were monitored using an electrocardiograph, with expired gases determined by an automated breath-by-breath system (Jaeger Oxycon ProTM, CareFusion, Höchberg, Germany). The parameters evaluated were: maximum/peak absolute and relative oxygen consumption (VO2 max), maximum heart rate and heart rate at ventilatory threshold 2 (VT2) using the model described by Skinner and Mclellan [27] for VT2 calculation. All measures were analyzed using software (LABManager 5.3.0.4, VIASYS Healthcare GmbH, Höchberg, Germany) and were stored in a personal computer for later recall.

#### 2.4.2. Exercise-Induced Oxidative Stress Cycling Protocol

In this test, subjects performed a SWEET test on the same device but with a constant load equivalent to a speed corresponding to 75% of its relative VO2 max calculated in the preliminary IETE test maintaining the same 2% slope. Subjects whose heart rate at 75% VO2 max exceeded the heart rate at VT2 (which means the exercise at this intensity would be more anaerobic and could be too exhausting to be maintained for 90 min) performed the trial 5 bmp lower than their heart rate at VT2. The duration of the test was 90 min, with ad libitum water consumption during the test. Subjects were weighed immediately before and after the test. The water consumption protocol was as follows: At the beginning of the test, the subject was given a bottle with 500 mL of mineral water (without gas) filled with a ml graduated cylinder. Subjects were provided as many bottles as requested. At the end of the test, the supplied bottles were counted by subtracting the water that was left in the last unit after measuring it with the graduated probe. Heart rate was monitored and recorded at 30, 60, and 90 min.

### 2.5. Biochemical Analysis and Markers

#### 2.5.1. Endogenous Antioxidant Effect of rDHA

The endogenous antioxidant effect of rDHA was determined by the difference of the oxidative stress as 8-hydroxy-2′-deoxyguanosine (8-OHdG) levels in 24-h urine samples in relation to weight by each cycling test before and after supplementation. Oxidative stress from each physical test was quantified by subtracting the resting (basal) values of 8-OHdG from the values after the SWEET on each occasion. Subjects collected a 24-h urine sample on the day before the cycling test and on the same day starting after the SWEET. If the sample was considered incomplete (some urination not collected), it was discarded from the study. After quantification of the total volume of urine excreted, a sample of 15 mL of each container was obtained and frozen at −80 °C for later analysis. The method used for this determination was the enzyme-linked immunosorbent assay (ELISA) with a kit called DNA Damage ELISA Kit (Catalog number #K059-H1, Assay Designs, Inc., Ann Arbor, MI, USA). The linearity of the kit was of R^2^ = 0.9956 and tests were performed thrice. Results were discarded and the assay repeated if the coefficient of variation (CV) between three tests was higher than 10%. This is a brief description of the method of the kit (for a full description, see [28]): A standard curve was generated with the 8-hydroxy-2′-deoxyguanosine (8-OHdG) stock solution provided. An 8-hydroxyguanosine conjugate was added to the standards and samples in each well, and binding initiated by peroxidase-labeled mouse monoclonal antibody. After a 2-h incubation, the plate was washed, and substrate added, which reacts with the peroxidase-labeled antibody that reacted with the bound conjugate. After a short incubation, the reaction was stopped, and the intensity of the generated color was detected in a microtiter plate reader capable of measuring a 450-nm wavelength. The concentration of the 8-hydroxy-2′-deoxyguanosine in the sample was calculated, after making suitable correction for the dilution of the sample.

Although more than 20 DNA damage of different bases are currently identified, only a small number of them were thoroughly studied, and 8-OHdG is the most certain considered. 8-OHdG appears in the DNA as a result of the damage by free radicals and can be extracted by repairing enzymes (8-hydroxy-2′-deoxyguanosine). If such repair does not occur, a base change (guanine for thymine) is induced during DNA replication. The cleaved 8-OHdG is then excreted in the urine [29]. However, not all 8-OHdG excreted in eliminated urine comes from oxidative stress to DNA and its subsequent repair; other factors like diet and cell death can modify such excretion. In the absence of these confounding factors, the urinary excretion of this metabolite should be attributed entirely to the repair of damaged DNA, so 8-OHdG can be considered as a marker for endogenous oxidative stress to DNA, a factor of initiation and promotion of carcinogenesis, and a risk factor for many diseases [30].

#### 2.5.2. DHA and PUFA Incorporation to Erythrocyte

Analysis of the fatty acid profile in the erythrocyte membrane was analyzed as follows: 10-mL blood samples were taken from the antecubital vein 10 min before the physical tests in tubes with tripotassium EDTA (ethylenediamine tetraacetic acid) washed with isotonic saline and purged with nitrogen gas and centrifuged at 2000 rpm for 30 min at 4 °C. The supernatant was collected and frozen at −80 °C and the cell phase (erythrocytes) was frozen at −80 °C for fatty acid analysis. Erythrocyte lipids were determined as methyl esters after methylation reaction, using the method of Lepage and Roy [31]. GC analysis was performed on a Shimadzu GCMS-QP2010 Plus gas chromatograph/mass spectrometer (Shimadzu, Kyoto, Japan). Fatty acid methyl esters (FAMEs) were identified through mass spectrometry and through comparison of the elution pattern and relative retention times of FAMEs with the reference FAME mixture (GLC-744 Nu-Chek Prep. Inc., Elysian, MN, USA). The results were expressed in relative amounts (percentage molar of total fatty acids) [32]. The fatty acid methyl esters are analyzed by capillary gas chromatography with an ionized flame detector [33].

### 2.6. Statistical Analysis

Quantitative variables are described as the mean with standard deviation. This description was made for the total sample and was stratified by the randomized treatment arm. Qualitative variables are presented in tabular form, including the relative and absolute frequencies for the treatment groups and the global sample. Data were checked prior to analysis; in all cases, the Kolmogorov–Smirnov test was employed to test for normal distribution and Levene′s test was used to test for homoscedasticity. The evolution of these quantitative variables was analyzed by parametric tests: a two-way repeated measures ANOVA test with one within-subject factor (product) and one between-subject factor (time) for the variables obtained in the SWEET endurance exercise test. For the post hoc group comparison, the Bonferroni test was employed. Statistical analysis was performed using SPSS software (v21.0, Chicago, IL, USA) and p values are reported for every group and group × time interaction; *p* < 0.05 is considered statistically significant.

Sample size was calculated according to the primary variable of 8-OHdG in urine. Taking into consideration the standard deviation of 105 ng of a previous study [34] and a desired precision of 100 ng, with an alpha risk of 5% and a statistical power of 80%, we required 14 participants in each group. We estimated a 10% dropout in each group, so a total of 15 participants per study arm was required.

## 3. Results

### 3.1. Participant Flow Diagram and Baseline Characteristics

**Figure 1:**
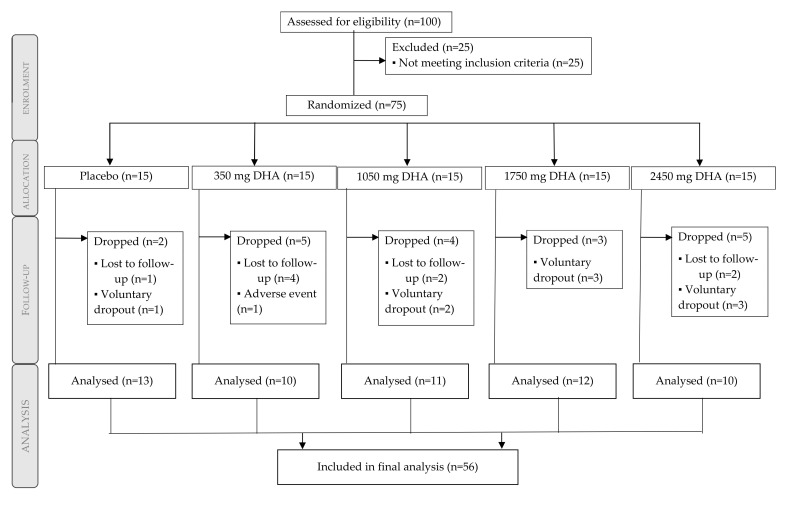
Participant flow diagram.

Fifty-six males were included in the final analysis (Figure 1). From the 75 participants randomized and balanced throughout the 5 groups (*n* = 15), a total of 19 participants dropped out: nine decided to voluntarily quit the study (no reason was provided), eight were lost in the follow-up, and one presented an adverse event (reflux), which was expected for the consumption of fish oils. Subjects completing the study according to the treatment group were 13 subjects (placebo), 10 subjects (350 mg/day rDHA), 11 subjects (1050 mg/day rDHA), 12 subjects (1750 mg/day rDHA), and 10 subjects (2450 mg/day rDHA).

Demographic characteristics of subjects are presented in Table 1. Either age (38.6 ± 9.8 years; mean ± SE), weekly training time and cycling (*p* = 0.961 and *p* = 0.518 respectively), training conditioning as per their estimated relative maximum oxygen consumption (*p* = 0.536), and weight (*p* = 0.867) were similar between groups. Therefore, we assumed homogeneity of the different study groups. Detailed information about the absolute and relative oxygen consumption can be found in Appendix A, Table A2.

### 3.2. Physical Tests

All groups significantly increased the excretion of 8-OHdG after the first cycling test (placebo *p* < 0.001; 350 mg *p* = 0.008; 1050 mg *p* = 0.002; 1750 mg *p* < 0.001; 2450 mg *p* = 0.003), thus we assumed an effective exercise-induced oxidative stress among all groups by this physical test.

Environmental conditions (humidity and temperature) were similar between the two occasions (*p* = 0.985 for humidity and *p* = 0.265 for temperature) and along the different groups (*p* = 0.967 for humidity and *p* = 0.863 for temperature). Therefore, we assumed that environmental conditions were homogeneous over time between all groups.

The hydration assessment showed no significant differences on water consumption during the SWEET on each occasion (*p* = 0.454, for numerical data, see Appendix A, Table A3). Not surprisingly, subjects in all groups suffered weight loss due to sweating after the test (*p* = 0.002) on each occasion, with a mean weight reduction of 0.9%. Additional information about the course of the heart rate during the trial can be found in Appendix A, Table A4.

### 3.3. Nutritional Assessment

All groups had a similar intake for omega-3 (*p* = 0.651), omega-6 (*p* = 0.256), and PUFA (*p* = 0.770). The antioxidant micronutrients vitamin C (*p* = 0.905) and E (*p* = 0.794) and zinc (*p* = 0.927) as well as energy intake (*p* = 0.745) were also not different among groups. More details for each group can be found in Table 2.

### 3.4. Endogenous Antioxidant Effect of rDHA

**Figure 2:**
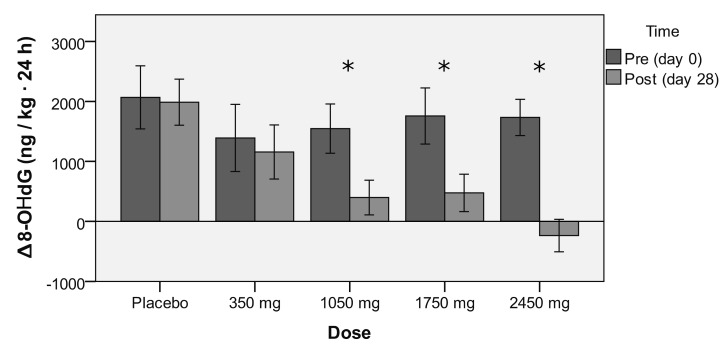
Comparison of ∆8-OHdG (difference of 8-OHdG 24-h urine excretion before and after the constant load cycling test (SWEET)) relative to weight, among the placebo and rDHA groups (with increasing doses), prior to (Pre: Day 0) and post (Post: Day 28) supplementation. Values are presented as mean ± standard error (SE). * *p* < 0.05.

Data shows a dose–response effect from rDHA against exercise-induced oxidative stress (Table 3, *p* = 0.026; Placebo F = 0.041; 350 mg F = 0.268; 1050 mg F = 7.112; 1750 mg F = 9.681; 2450 mg F = 15.230), which reached statistical significance for doses equal to or greater than 1050 mg/day (Figure 2, Placebo *p* = 0.841; 350 mg *p* = 0.607; 1050 mg *p* = 0.010; 1750 mg *p* = 0.003; 2450 mg *p* < 0.001), with a trend towards neutralization at the dose of 2450 mg/day. Supplementation did not change resting oxidative stress between the groups (*p* = 0.319) or within each group (*p* = 0.144).

### 3.5. DHA and PUFA Incorporation to Erythrocyte

**Figure 3:**
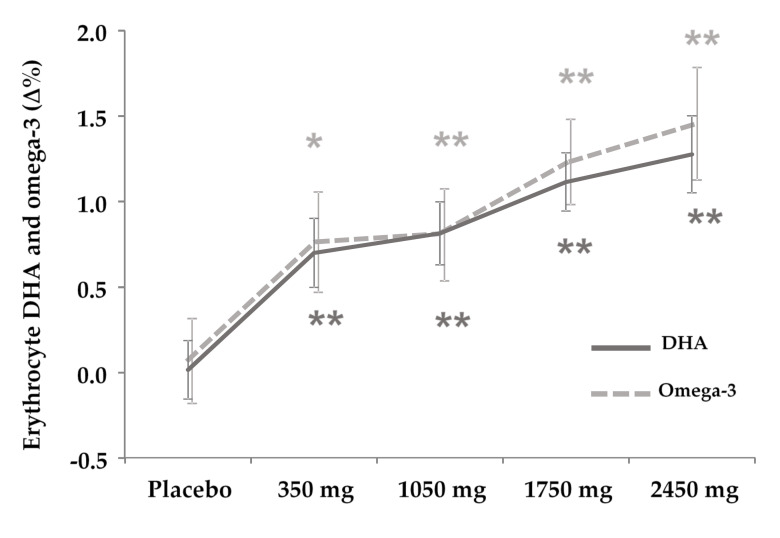
DHA and omega-3 composition in the erythrocyte membrane. Change is represented as the difference of percentages (∆%) from baseline conditions within each group. Values are presented as mean ± standard deviation. * *p* < 0.05; ** *p* < 0.01 statistically significant compared to baseline within each group.

The fatty acid profile in the cell membrane of erythrocytes (Table 4) showed a significant increase in the rDHA groups in a dose-dependent manner for rDHA (350 mg *p* < 0.001, 1050 mg *p* < 0.001, 1750 mg *p* < 0.001, 2450 mg *p* < 0.001, Figure 3) and omega-3 (350 mg *p* = 0.012; 1050 mg *p* = 0.004; 1750 mg *p* < 0.001; 2450 mg *p* < 0.001, Figure 3), and showed a decrease also in a dose-dependent manner in omega-6 from 1050 mg but not for 2450 mg (1050 mg *p* = 0.050; 1750 mg *p* = 0.028; 2450 mg *p* = 0.091) and the omega-6 to omega-3 ratio from 350 mg (350 mg *p* = 0.018; 1050 mg *p* = 0.009; 1750 mg *p* < 0.001; 2450 mg *p* < 0.001). EPA and total PUFA remained unchanged.

## 4. Discussion

### 4.1. Main Finding: rDHA Reduces Constant-Load Medium-Intensity Aerobic Exercise-Induced Oxidative Stress Dose-Dependently

In the present study, we found that supplementation for 4 weeks (28 days) of a re-esterified DHA counteracted exercise-induced oxidative stress (generated by a constant-load aerobic exercise) from 1050 mg a day, in a dose-dependent manner, with a trend towards neutralization at the highest dose of 2450 mg. This finding can be useful to prescribe supplementation strategies that aim to decrease oxidative stress, in which a dose-dependent response is expected to occur. With this purpose, we also suggest taking into account the following factors: (a) Total cumulative dose (total grams during the treatment); (b) standardization (purity and total DHA content); (c) chemical structure and configuration of the product (triglyceride, etyl-esther or re-esterified form); and (d) days of treatment (since some adaptations may require structural conformation, which might not be accelerated by a higher daily dose).

To the best of our knowledge, this is the first trial in humans examining the dose–response effect of a re-esterified DHA against moderate-intensity exercise-induced oxidation. Contrary to our results, a previous study found that supplementation with omega-3 fish oil (400 mg of DHA and 2000 mg EPA/day for 6 weeks) increased exercise-induced oxidative stress plasma markers (as F2-isoprostanes [35]) in people engaged in routine cycling (about 1 h/day) after a 3-h cycling bout (at 57% of maximum watts) for three consecutive days [36]. In contrast, urinary F2-isoprostanes were reduced by omega-3 consumption in combination with aerobic exercise [20] either as fish oil (4 g/day of purified DHA or EPA, for 6 weeks) in mildly hyperlipemic overweight men without exercise prescription [37] or as dietary fish meals (3.6 g ω-3/day for 8 weeks) in type 2 diabetes mellitus, engaged in moderate (55–65% VO_2_max) or light (heart rate <100 bpm) exercise [38]. However, F2-isoprostanes derive solely from the oxidation of arachidonate [39], which are lipid peroxidation by-products, and therefore not a general marker of oxidative stress to cells (like 8-oxo-dG), as was the case in this experiment. Additionally, F2-isoprostanes along with other oxidation by-products of DHA promise further research due to its presumably anticancer properties [40], so interpretation of this marker as an undesirable outcome from oxidative stress should be carefully considered.

The product employed in this trial is different from regular fish oils, for instance, because it is predominantly DHA over EPA. One possible advantage of DHA as an antioxidant and compared to EPA is that it preferentially accumulates in mitochondrial cardiolipin [41] and into phospholipids of the cell membrane, where they can exert antioxidant properties, different to dietary antioxidants, which act as a barrier to external oxidation, which interacts with endogenous antioxidants to form a cooperative network of cellular antioxidants [42], thus providing a different antioxidant defense system. Apart from modifying tissue composition, DHA is capable of stimulating endogenous antioxidant systems by the activation of signaling pathways to upregulate intracellular endogenous antioxidants (MnSOD or SOD2: a mitochondrial form of SOD), probably as a side mechanism to protect the new cell’s membrane composition more prone to oxidation due to double chains in PUFAs [43], which may also be a mechanism that contributes to an organism’s antioxidant defense system. Achieving such a dose of DHA daily by dietary intake of fish products can be more difficult than taking a DHA food supplement regularly, especially in areas with low availability of fish products, and to a broader population if they are too expensive for them. Regular fish oils represent an alternative but usually lack standardization in their active components like DHA, which are subject to the natural variability of food by-products. To this respect, the re-esterified DHA product employed in this study can assure homogeneity in their composition and guarantee proper administration.

One limitation of the study was the low number of participants that were finally analyzed. This was due to a higher drop-out rate than the 10% we expected, which perhaps was too optimistic. Another limitation was the blinding of subjects since subjects receiving one softgel (350 mg group) would guess they belonged to the low-dose group, and those receiving seven softgels (2450 mg group) would guess they belonged to the high-dose group. Subjects did not belong to the same social group (i.e., same cycling team) so communication between participants to guess the number of softgels in the intermediate- and high-dose groups as well as the control product was partially impeded during the study. To overcome this problem, pill count-matched controls would be employed but would inevitably increase study cost.

### 4.2. Secondary Finding: DHA Is Incorporated to Erythrocytes Dose-Dependently

As a secondary finding, we found dose-dependent incorporation of rDHA to erythrocyte cells in all groups (starting from 350 mg/day) after 4 weeks of supplementation, which was paralleled by an increase in the overall omega-3 percentage, and a change in the omega-6/omega-3 ratio. This is in line with previous studies [44], which might be favored by the modified triacylglycerol composition of the product employed (re-esterified (rTAG), instead of the etyl-ester forms, more widely employed in the literature) partly due to placement of DHA in the second position (sn-2) of the triacylglycerol [45], which is the monoglyceride position preserved after pancreatic lipase action during the digestion process [21]. To this respect, a previous clinical trial, divided in two studies [46,47], found that 3 and 6 months of supplementation with rTAG increased the DHA and omega-3 index of the FA composition of erythrocytes in a more pronounced manner in the rTAG group compared to the etyl-ester (EE) group (and both compared to placebo). In the present study, we found this outcome with a shorter supplementation time (4 weeks). One explanation can be that our product was richer in DHA while the previous trial employed primarily EPA, which requires transformation to DHA by the enzyme omega-3 desaturase (which adds the additional double bond required to the C15) which showed variability in its conversion to DHA in previous clinical trials [48]. Previous studies found that DHA incorporation to other tissues is paralleled by incorporation of DHA to erythrocyte tissues [49,50], thus we assumed that the product employed effectively reached target tissues to exert antioxidant properties.

### 4.3. Contextualization of the Findings

The capacity of DHA to act as an antioxidant must be discussed in a broader sense to understand the current findings. As discussed earlier, blood plasma contains circulating compounds (like vitamins and polyphenols), which can exert antioxidant properties, acting as first barrier agents. In this study, we controlled and quantified dietary intake of these compounds, but as a limitation of the study, we did not measure plasma oxidation biomarkers or endogenous antioxidant enzymes to ascertain whether the antioxidant property of rDHA was due to a barrier effect in plasma or due to an enhancement of the intracellular endogenous antioxidant defense, which is the expected antioxidant effect of DHA [43]. Therefore, we examined the capacity of DHA to prevent oxidative stress to cells nonspecifically and should not be considered as the only strategy to increase antioxidant defenses against exercise-induced oxidation. Likewise, the model employed showed decreased oxidative stress in a specific condition with dietary control (limiting antioxidant and PUFA intake), in a narrow population sample (males engaged in regular cycling) subject to regular exercise-induced oxidative stress (with an adaptation of their endogenous oxidative defense system). The generalizability of these findings is limited and should not be assumed to be reproduced in a general population without control of dietary antioxidants or previous adaptation to exercise-induced oxidative stress by regular physical training. However, we think that the dose–response effect is expected to be reproduced in a broader population.

In a broader context, employment of exogenous antioxidants can interfere with exercise training-induced adaptations, which require signaling activation mediated by exercise-induced oxidative stress [51,52], to the point that some authors consider they have more harm than good in health and sports [53,54]. On the other hand, oxidative stress is increased without any benefit in some chronic diseases or physiopathological conditions, or as a consequence of their concomitant pharmacological treatment in which adjuvant treatment with exogenous antioxidants may be beneficial [55]. They conform a broad family of substances and molecules like vitamins [56] and polyphenols [57,58], which usually act as first barrier agents, while DHA seems to act as a pro-oxidant, which can increase endogenous enzymatic antioxidant defenses [9,10,14]. Both kinds of exogenous and endogenous antioxidant systems interact in a cooperative network of cellular antioxidants [42], with different underpinning antioxidative mechanisms acting at different sites in the body and cell. Therefore, recommendations about the employment of exogenous antioxidants and/or increasing of endogenous antioxidant systems and overall prevention of oxidative stress in sport and health require a thorough study in each circumstance, the discussion of which is extensive and out of the scope of this work.

## 5. Conclusions

Supplementation of re-esterified DHA exerts a dose-dependent antioxidant property against moderate-intensity long-duration aerobic exercise in physically active subjects when provided at a dose of at least 1050 mg a day for 4 weeks.

## Figures and Tables

**Table 1:** Demographic characteristics of subjects. Values are presented as mean ± standard deviation. Statistically significant *p* < 0.05.

Group	Age (Years)	Training Time (h)	Cycling (h)	VO2 Max (mL/min kg)	Weight (kg)
Placebo	38.6 ± 12.2	8.3 ± 7.3	6.3 ± 3.8	45.1 ± 5.6	75.8 ± 9.3
350 mg	36.9 ± 5.6	9.7 ± 2.0	8.5 ± 2.5	48.2 ± 5.2	74.9 ± 5.7
1050 mg	37.8 ± 11.2	9.1 ± 2.7	6.9 ± 2.2	45.0 ± 5.7	76.7 ± 5.9
1750 mg	40.9 ± 8.9	9.2 ± 5.1	8.5 ± 5.3	47.2 ± 9.7	76.3 ± 5.0
2450 mg	38.4 ± 10.4	9.6 ± 3.5	8.0 ± 3.9	43.1 ± 6.9	79.9 ± 12.7
ANOVA *p*-value	0.909	0.961	0.518	0.536	0.867

**Table 2:** Nutritional assessment of the diet during the intervention. Values are presented as mean ± standard deviation. Statistically significant *p* < 0.05.

Group	Energy (Kcal)	Vitamin C (mg)	Vitamin E (mg)	Zinc (mg)	PUFA (g)	Omega-6 (g)	Omega-3 (g)
Placebo	2512 ± 685	513 ± 490	8.1 ± 11.4	8.1 ± 10.9	15.5 ± 9.3	4.1 ± 2.0	1.1 ± 1.1
350 mg	2302 ± 627	396 ± 550	6.4 ± 6.4	10.5 ± 7.9	18.6 ± 18.7	5.8 ± 3.5	1.0 ± 1.1
1050 mg	2646 ± 837	336 ± 204	5.0 ± 5.5	8.5 ± 6.3	14.2 ± 9.1	4.5 ± 3.4	1.4 ± 1.4
1750 mg	2731 ± 916	463 ± 464	7.2 ± 11.5	10.8 ± 8.8	15.9 ± 7.0	3.6 ± 1.9	1.8 ± 1.3
2450 mg	2484 ± 356	392 ± 395	7.5 ± 5.5	9.5 ± 7.5	11.8 ± 3.8	3.1 ± 0.8	0.9 ± 0.8
ANOVA *p*-value	0.745	0.905	0.794	0.927	0.770	0.256	0.651

PUFA: polyunsaturated fatty acids.

**Table 3:** rDHA relative antioxidant effect after the SWEET (constant load cycling). Values are presented as mean ± standard deviation. Statistically significant * *p* < 0.05; ** *p* < 0.01.

Group	Time	Basal (ng/kg at 24 h)	Oxidative Stress (ng/kg at 24 h)	rDHA Antioxidant Effect (F-Snedecor)	ANOVA (Ox. Damage Time)
Placebo	Day 0	3965 ± 388	2067 ± 1894 **	80 ± 1400 (F = 0.041)	*p* = 0.026
	Day 28	4233 ± 453	1987 ± 1387 **		
350 mg	Day 0	4084 ± 442	1391 ± 1768 **	234 ± 1526 (F = 0.268)	
	Day 28	4388 ± 517	1157 ± 1428 **		
1050 mg	Day 0	3328 ± 422	1546 ± 1360 **	1148 ± 1690 * (F = 7.112)	
	Day 28	3418 ± 493	398 ± 959		
1750 mg	Day 0	3924 ± 404	1757 ± 1621 **	1282 ± 1247 ** (F = 9.681)	
	Day 28	4119 ± 472	475 ± 1083		
2450 mg	Day 0	4352 ± 494	1734 ± 854 **	1970 ± 1179 ** (F = 15.230)	
	Day 28	4472 ± 578	−236 ± 765		

Basal: 8-OHdG 24-h urine excretion in relation to weight (ng/kg 24 h) before the cycling test. Oxidative stress (as a result of the SWEET): 8-OHdG 24-h urine excretion in relation to weight—basal values). rDHA antioxidant effect: oxidative stress at day 28 minus oxidative stress at day 0 expressed as effect size (F-Snedecor).

**Table 4:** Erythrocyte polyunsaturated fatty acid (PUFA) composition (percentage of total fatty acids) at baseline (day 0) and after rDHA supplementation (day 28). Values are presented as mean ± standard deviation. * *p* < 0.05; ** *p* < 0.01 statistically significant compared to baseline within each group.

Group	Time	PUFA	Omega-3	Omega-6	n-6/n-3 Ratio	DHA	EPA
Placebo	Day 0	32.2 ± 1.4	5.6 ± 0.9	26.7 ± 1.1	4.9 ± 0.8	3.7 ± 0.6	0.46 ± 0.22
	Day 28	32.4 ± 2.0	5.6 ± 1.1	27.0 ± 1.3	5.0 ± 1.0	3.7 ± 0.7	0.45 ± 0.17
350 mg	Day 0	32.3 ± 1.2	5.9 ± 1.6	26.4 ± 1.5	4.8 ± 1.4	3.9 ± 1.1	0.53 ± 0.23
	Day 28	32.3 ± 3.8	6.7 ± 1.6 *	25.7 ± 3.3	4.0 ± 1.0 *	4.6 ± 1.2 **	0.56 ± 0.21
1050 mg	Day 0	32.1 ± 1.3	5.8 ± 1.8	26.4 ± 2.6	5.0 ± 1.7	3.7 ± 0.8	0.70 ± 0.71
	Day 28	32.0 ± 1.5	6.6 ± 1.9 **	25.4 ± 2.3 *	4.2 ± 1.4 **	4.5 ± 0.9 **	0.81 ± 0.60
1750 mg	Day 0	32.3 ± 1.2	5.4 ± 1.2	26.9 ± 1.3	5.4 ± 1.8	3.6 ± 0.8	0.44 ± 0.23
	Day 28	32.5 ± 1.4	6.6 ± 1.0 **	25.9 ± 1.4 *	4.0 ± 0.9 **	4.7 ± 0.8 **	0.64 ± 0.20
2450 mg	Day 0	32.9 ± 1.1	6.2 ± 1.5	26.7 ± 1.5	4.7 ± 2.1	4.2 ± 1.0	0.51 ± 0.26
	Day 28	33.3 ± 1.4	7.7 ± 1.2 **	25.7 ± 1.5	3.5 ± 0.9 **	5.4 ± 0.9 **	0.72 ± 0.15

## Appendix A

**Table A1:** List of products with antioxidant properties that should not be consumed at breakfast prior to the test.

Fruits	Vegetables	Grain	Oils	Animal Products
Strawberries	Pepper	Coffee	Seed oils	Fish
Raspberries	Carrot	Green Tea	Fish oils	
Raspberries	Tomato	Cocoa	Seaweed oils	
Grapes	Lettuce	Cereals		
Kiwis	Spinach	Corn		
Blueberries	Onion	Nuts		
Plums	Garlic	Walnuts		
Orange		Soy and derivates		
Lemon		Wheat germs		
Papaya				
Apples				
Pineapple				
Avocado				
Red wine				

**Table A2:** Maximum oxygen consumption and oxygen consumption at ventilatory threshold 2 (absolute and relative to weight) for each of the study groups, expressed both as absolute (mL per minute) and relative to weight (mL per minute and kg). Values are presented as mean ± standard deviation.

Group	VO2 Max mL/min	VO2 Max mL/min kg	VT 2 mL/min	VT 2 mL/min kg
Placebo	3390.9 ± 354.9	45.1 ± 5.6	2559.5 ± 296.7	34.2 ± 5.2
350 mg	3607.7 ± 439.5	48.2 ± 5.2	2702.2 ± 326.9	36.2 ± 4.8
1050 mg	3430.5 ± 274.5	45.0 ± 5.7	2666.5 ± 423.3	34.9 ± 5.7
1750 mg	3590.0 ± 728.8	47.2 ± 9.7	2737.6 ± 452.0	35.9 ± 6.2
2450 mg	3417.9 ± 602.5	43.1 ± 6.9	2873.9 ± 370.3	36.4 ± 5.1
Total	3487.4 ± 492.5	45.8 ± 6.8	2693.9 ± 377.2	35.4 ± 5.3

**Table A3:** Water consumption during the SWEET test. Values are expressed as mean ± standard deviation.

Group	Trial	Water Consumption (mL)
Placebo	Day 0	1043.8 ± 756.5
	Day 28	948.1 ± 599.5
350 mg	Day 0	590.0 ± 307.1
	Day 28	791.0 ± 535.4
1050 mg	Day 0	601.8 ± 358.7
	Day 28	680.0 ± 417.4
1750 mg	Day 0	967.1 ± 550.2
	Day 28	1214.2 ± 642.5
2450 mg	Day 0	568.8 ± 243.4
	Day 28	400.0 ± 181.3

**Table A4:** Heart rate during the SWEET at 30, 60, and 90 min, at baseline (day 0) and after intervention (day 28). Values are presented as mean beats per minute (bpm) with standard deviation.

Group	Trial	Time	Heart Rate (bpm)
Placebo	Day 0	30 min	157.9 ± 11.5
		60 min	154.7 ± 12.5
		90 min	155.7 ± 10.4
	Day 28	30 min	154.7 ± 12.1
		60 min	154.9 ± 10.7
		90 min	155.1 ± 8.4
350 mg	Day 0	30 min	152.8 ± 8.6
		60 min	152.8 ± 8.0
		90 min	149.9 ± 7.1
	Day 28	30 min	151.3 ± 8.2
		60 min	150.1 ± 9.1
		90 min	155.8 ± 14.9
1050 mg	Day 0	30 min	158.1 ± 15.4
		60 min	158.1 ± 13.8
		90 min	157.8 ± 7.4
	Day 28	30 min	157.4 ± 10.9
		60 min	157.0 ± 11.0
		90 min	157.9 ± 11.5
1750 mg	Day 0	30 min	154.7 ± 12.5
		60 min	155.7 ± 10.4
		90 min	154.7 ± 12.1
	Day 28	30 min	154.9 ± 10.7
		60 min	155.1 ± 8.4
		90 min	152.8 ± 8.6
2450 mg	Day 0	30 min	149.9 ± 7.1
		60 min	151.3 ± 8.2
		90 min	150.1 ± 9.1
	Day 28	30 min	155.8 ± 14.9
		60 min	158.1 ± 15.4
		90 min	158.1 ± 13.8
