# Correction: de Salazar et al. Oxidative Stress in Endurance Cycling Is Reduced Dose-Dependently after One Month of Re-Esterified DHA Supplementation. *Antioxidants* 2020, *9*, 1145

**DOI:** 10.3390/antiox11030449

**Published:** 2022-02-24

**Authors:** Lydia de Salazar, Carlos Contreras, Antonio Torregrosa-García, Antonio J. Luque-Rubia, Vicente Ávila-Gandía, Joan Carles Domingo, Francisco Javier López-Román

**Affiliations:** 1 Sports Physiology Department, Faculty of Health Sciences, UCAM Universidad Católica San Antonio de Murcia, 30107 Murcia, Spain; dssflydia@ucam.edu (L.d.S.); ajluque@ucam.edu (A.J.L.-R.); vavila@ucam.edu (V.Á.-G.); jlroman@ucam.edu (F.J.L.-R.); 2 Innova, Health and Sport Institute, 30100 Murcia, Spain; cjcontreras@ucam.edu; 3 Department of Biochemistry and Molecular Biomedicine, University of Barcelona, 08007 Barcelona, Spain; jcdomingo@ub.edu; 4 Biomedical Research Institute of Murcia (IMIB-Arrixaca), 30120 Murcia, Spain

**Carlos Contreras** was not included as an author in the published article [1]. The corrected Author Contributions Statement appears here.

The contributions as an author were Investigation and Resources.

The affiliation is Innova, Health and Sport Institute, 30100 Murcia, Spain, and e-mail is cjcontreras@ucam.edu.

The authors apologize for any inconvenience caused and state that the scientific conclusions are unaffected. The original article has been updated.
